# Racial Disparity in the Associations of Cotinine with Insulin Secretion: Data from the National Health and Nutrition Examination Survey, 2007-2012

**DOI:** 10.1371/journal.pone.0167260

**Published:** 2016-12-19

**Authors:** Rong Liu, Zheng Zheng, Jie Du, Katherine Kaufer Christoffel, Xin Liu

**Affiliations:** 1 Department of Epidemiology, Beijing Anzhen Hospital, Capital Medical University, Beijing Institute of Heart, Lung and Blood Vessel Diseases, Beijing, China; 2 Department of Neurobiology, Beijing Institute of Geriatrics, Beijing Xuan Wu Hospital, Capital Medical University, Beijing, China; 3 Beijing An Zhen Hospital, Capital Medical University, The Key Laboratory of Remodeling-related Cardiovascular Diseases, Ministry of Education, Beijing Institute of Heart, Lung and Blood Vessel Diseases, Beijing, China; 4 Mary Ann and J. Milburn Smith Child Health Research Program, Department of Pediatrics, Northwestern University Feinberg School of Medicine, Chicago, IL, United States of America; 5 Key Laboratory of Genomic and Precision Medicine, China Gastrointestinal Cancer Research Center, Beijing Institute of Genomics, Chinese Academy of Sciences, Beijing, China; 6 Department of Preventive Medicine, Northwestern University Feinberg School of Medicine, Chicago, IL, United States of America; 7 University of Chinese Academy of Sciences, Beijing, China; Chinese Academy of Medical Sciences, CHINA

## Abstract

**Background:**

Although relationships between smoking/high cotinine and type 2 diabetes have consistently been observed, few studies have investigated the relationship between cotinine and underlying pathophysiological defects that characterize diabetes aetiology. This study aimed to test the associations between cotinine and measures of insulin resistance or insulin secretion.

**Methods:**

This analysis included 5,751 non-diabetic adult American from the National Health and Nutrition Examination Survey (NHANES) from 2007–2012. Insulin function was represented with two indexes: insulin resistance index (HOMA-IR) and insulin secretion index (HOMA-B) estimated by homeostasis model assessment. We categorized cotinine levels into quartiles and estimated the odds of HOMA-IR in the 4^th^ quartile and HOMA-B in the 1^st^ quartile among cotinine categories using multiple logistic regression models.

**Results:**

Cotinine concentration was not associated with the risk of high HOMA-IR. Association of cotinine with low HOMA-B existed and differed by race/ethnicity (P for interaction<0.05). High cotinine concentration (in the 4^th^ quartile) was associated with an increased risk of low HOMA-B compared with low cotinine concentrations(1^st^ -2^nd^ quartiles) among white (odds ratio[OR], 1.51 [95% confidence interval[CI], 1.16–1.97]) or black participants (OR, 2.98 [95%CI, 1.90–4.69]) but not among Mexican (OR, 1.79 [95%CI, 0.90–3.53]) or other Hispanic(OR, 1.02 [95%CI, 0.56–1.86]) participants. Such associations remained significant even after further adjustment for HOMA-IR.

**Conclusions:**

High cotinine is associated with decreased insulin secretion function only in white and black non-diabetic U.S. adult population. Results evaluating cotinine in ethnically homogeneous populations may not be broadly generalizable to other racial/ethnic groups.

## Introduction

Type 2 diabetes is epidemic. In the U.S., there are about 1.5 million new cases per year, and the crude prevalence of diabetes (diagnosed plus undiagnosed) is reported at 9.6% in adults [1]. Cigarette smoking is another global public health concern, causing the death of about 4 million people every year[2]. A number of previous studies[3] have assessed the association between smoking and the incidence of type of diabetes, suggesting that active smoking could be involved in the development in the glucose abnormalities. Since impaired insulin secretion and insulin resistance are the main pathophysiological components of type 2 diabetes, these two defects are likely be the potential mechanism underlying the smoking-diabetes linkage. Although a few of population-studies [4–7] have investigated the association of smoking and insulin, the conclusions remain controversial.

Cotinine is a major metabolite of nicotine that is used as a marker for both active smoking and tobacco smoke exposure ("passive smoking")[8]. Cotinine is generally preferred over nicotine for such assessments because of its substantially longer half-life[9]. Using cotinine would minimize the bias if smokers do not accurately report their smoking status. A recent U.S. study[10] in a non-diabetic sample reported that both cotinine and self-reported smoking were associated with increased glycated hemoglobin A1c (HbA1c). However, little has been known regarding the role of insulin resistance and β cell function in the linkage of smoking/cotinine and hyperglycemia in large non-diabetic population.

We conducted this study to assess the association of cotinine concentration with insulin resistance and insulin secretion function, using data from the National Health and Nutrition Examination Survey (NHANES) 2007–2012. Since the metabolism of cotinine vary substantially by race/ethnicity[11], we were particularly interested in whether these associations, if exist, differ according to race/ethnicity.

## Material and Methods

### Study sample

**Fig 1:**
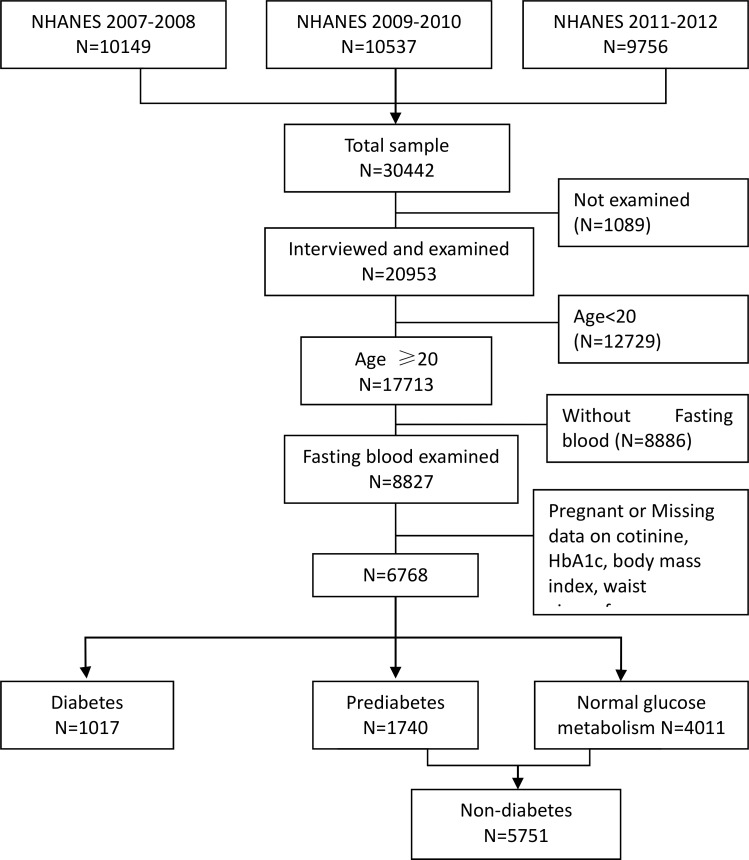
Flowchart depicting three successive waves (2007–2012) of continuous NHANES used for analysis.

The National Health and Nutrition Examination Survey (NHANES), conducted by the National Center for Health Statistics[12], was designed to be representative of the U.S. civilian non-institutionalized population using complex, multistage probability samples. The NHANES protocol was approved by the National Center for Health Statistics Ethics Review Board[13], and written informed consent was obtained from all participants. Participants were interviewed in homes and subsequently received a physical and laboratory examination in a mobile examination center. We combined three successive waves (2007–2012) of the continuous NHANES for our analysis, generating a total sample of 20,953 individuals. We limited the analysis to adult participants(n = 8,827) with age ≥20 years old, who completed fasting glucose(FPG) and fasting insulin(FPI), HbA1c and cotinine assessment. We further excluded subjects who were pregnant (n = 1,059) or with diabetes (n = 1,017), resulting a final sample size of 5,751 participants (Fig 1).

### Cotinine status

Cotinine is used as a biological surrogate for smoking and tabocco exposure. Serum cotinine was measured using isotope dilution-high performance liquid chromatography/atmospheric pressure chemical ionization tandem mass spectrometry [14]. We classified cotinine concentrations in all eligible participants into four groups according to quartile, 1^st^, 2^nd^, 3^rd^ and 4^th^ quartile or race/ethnic-specific quartile groups.

### Glycated hemoglobin, glucose and insulin

HbA1c was measured using high performance liquid chromatography. FPG and FPI were measured in participants examined after an 8-24-h fast, using the hexokinase enzymatic method. Due to the method of insulin assay 2007–2010 switched from Mercodia sandwich ELISA assay to Roche chimilumnescent immunoassay 2011–2012, we converted fasting insulin values from 2011–2012 to make them comparable to values from 2007–2010 in our analysis, using the formula suggested by NHANES[15]:

$$Insulin\;(Mercodia-equivalent)=0.6295+1.0770\times Insulin\;(Roche)-8.566\times 10^{-3}\times Insulin\;{\left(Roche\right)}^{2}$$

### Other covariates

Demographic information included age, race/ethnicity, and education. Race/ethnicity was categorized as non-Hispanic White, non-Hispanic Black, Mexican American and other Hispanic. Education level was classified as below high school, high school, and above high school. Weight and height were measured using standardized techniques and equipment during clinical examinations. Body mass index (BMI) was calculated as weight in kilograms divided by height in meters squared. Waist circumference (WC) was measured at the upper-most lateral border of the ilium. Alcohol consumption was dichotomized as 0, ≤1, 2–3, and ≥4 drinks per week. Physical activity was categorized into three levels (low, moderate and high) according to the total metabolic equivalent score from questionnaires.

### Outcomes of Interest

Homeostatic model assessment (HOMA) [16] is a method for assessing insulin resistance and insulin secretion estimated from fasting glucose and insulin or C-peptide concentrations. HOMA has been validated against a variety of physiologic methods and widely used in epidemiology and clinical studies[17]. Insulin resistance and insulin secretion index are calculated as:

HOMA−IR=FPI(mU/L)×FPG(mmol/L)÷22.5

$$HOMA-B=\frac{20\times FPI(mU/L)}{FPG(mmol/L)-3.5}$$

Since the studies on HOMA-IR index to detect impaired glucose tolerance or metabolic syndrome are limited and resulted in different cut offs. [18–21], and data on cut off of homeostasis model assessment for insulin secretion is scarce, we used HOMA-IR in top quartile to define the risk of insulin resistance, and HOMA-B in bottom quartile to define the risk of impaired insulin secretion.

### Statistical analysis

We accounted for the survey sampling design and used sample weights to generalize estimates to the U.S. population as a whole[12]:

$$Weight=\frac{1}{3}\times wtsaf2yr\;(\text{fating}\;\text{subsample}\;2\;\text{year}\;\text{weight})$$

for 2007–2012.

Participant characteristics were tested for differences across cotinine quartile categories. Differences in frequencies were examined by chi-squared tests for categorical variables. Differences in means were tested by ANOVA for continuous variables. To examine the functional forms of the association of cotinine with HOMA-IR and HOMA-B, we applied adjusted penalized smoothing splines. Logistic regression models were used to examine the relationship between cotinine and odds of HOMA-IR in 4^th^ quartile and HOMA-B in 1^st^ quartile. We first compared participants with cotinine in high (4^th^ quartile) and middle(3^rd^ quartile) groups with counterparts with cotinine in low(1^st^ -2^nd^ quartile) group. Second, we performed test for trend across categories of cotinine. Third, we investigated race/ethnicity–cotinine interaction by adding a product term for cotinine levels and race/ethnicity categories to the regression model. The base model included age, gender, race/ethnicity, education attainment and alcohol consumption. The second model added variables possibly cofounding or mediating the associations of interest, such as physical activity levels and waist circumference. Analyses stratified by race/ethnicity were conducted using the same models.

There were 767(11%) participants reporting antihypertensive (beta blocker, diuretic and/or vasodilator) use, which may exert influence on insulin secretion[22]. Sensitive analyses were conducted with further adjusted for antihypertensive use or with exclusion of these participants from the analyses.

Statistical tests were two-sided and P<0.05 was considered statistical significant. We applied GAM in R program (version 2.15.3, R Core Team, Vienna, Australia)[23] to fit spline model. We used survey procedures in SAS software (version 9.2, SAS Institute, Inc., Cary, North Carolina) to account for NHANES sampling design and sample weights.

## Results

After the exclusion of participants with diabetes either self-reported or diagnosed with HbA1c criteria, the final sample consisted of 5,751 individuals. Geometric mean (95%CI) of serum cotinine was 0.31(0.25–0.38) ng/mL and varied substantially by race/ethnicity: 0.33(0.25–0.44) ng/mL for white, 0.90(0.63–1.30) ng/mL for black, 0.10(0.07–0.14) ng/mL for Mexican, and 0.17(0.11–0.26) ng/mL for Hispanic. The characteristics of participants in this analysis are presented in Table 1. Gender and ethnicity differed across cotinine categories with Participants with higher proportions of female in lower cotinine categories and the lowest proportion of Mexican or other Hispanics in the highest cotinine category. BMI and WC also differed across cotinine categories with the highest mean BMI and WC in 3^rd^ quartile of cotinine. However, the highest mean HbA1c was observed in 4^th^ quartile of cotinine.

**Table 1:** Characteristics of adult participants without diabetes in NHANES 2007–2012 by cotinine categories.

		Cotinine categories			
Characteristic	Q1(n = 1496)	Q2(n = 1559)	Q3(n = 1540)	Q4 (= 1531)	P value
Age (yrs)	49.5 (0.5)	47.7(0.7)	43.3(0.7)	41.5(0.5)	<0.0001
BMI (kg/m^2^)	27.7 (0.2)	28.3(0.2)	29.2(0.3)	27.3(0.2)	<0.0001
WC (cm)	95.6 (0.5)	96.9(0.6)	99.1(0.7)	96.0(0.5)	<0.0001
FPG(mmol/L)	5.43(0.02)	5.53(0.02)	5.56(0.02)	5.50(0.02)	<0.0001
HbA1C(%)	5.38(0.01)	5.42(0.01)	5.39(0.02)	5.42(0.01)	0.0129
FPI(uU/mL)^a^	10.0(6.8–16.1)	10.5(6.9–16.9)	11.2(7.2–17.3)	9.9(5.9–16.0)	<0.0001
HOMA-IR^a^	2.4(1.6–4.0)	2.6(1.6–4.2)	2.7(1.7–4.3)	2.5(1.4–4.1)	<0.0001
HOMA-B(%)^a^	108(73–163)	108(73–163)	110(76–165)	103(66–155)	0.0051
Cotinine(ng/mL)^a^	0.01(0.01–0.01)	0.03(0.02–0.04)	0.11(0.07–0.25)	174.00(44.80–290.00)	—
Female,n(%)	892 (63)	820(56)	699(46)	562(39)	<0.0001
Education,n(%) < High school	268 (10)	320(13)	391(19)	482(28)	
High school	232(14)	287(18)	360(26)	419(30)	
> High school	925 (76)	879(69)	686(55)	502(42)	0.1575
Race/ethnicity, n(%) Non-Hispanic White	692 (75)	620(65)	612(65)	760(73)	
Non-Hispanic Black	145(5)	222(9)	323(14)	320(14)	
Mexican American	291(9)	278(10)	226(9)	118(4)	
Other Hispanic	196(6)	182(6)	149(6)	114(4)	
Alcohol consumption, n(%) None	502 (29)	568(31)	452(27)	265(17)	<0.0001
≤1 Drink/week	671(47)	645(45)	684(47)	736(52)	
2–3 Drinks/week	132(13)	140(12)	183(17)	214(17)	
≥4 Drinks/week	120(11)	133(11)	118(9)	188(14)	<0.0001
Physical Activity, n(%) None/light	579(35)	553(33)	500(30)	531(34)	
Moderate	542(42)	535(39)	473(37)	363(28)	
Vigorous	304(23)	398(28)	464(33)	509(38)	<0.0001

Data are presented as Mean (se), median (IQR) or N (%). BMI, body mass index. WC, waist circumference. HbA1C, Glycated hemoglobin A1c. FPG, fasting glucose. FPI, fasting insulin. HOMA-IR, insulin resistance estimated by homeostasis model assessment. HOMA-B, insulin secretion estimated by homeostasis model assessment.

^a^, log transformed in ANOVA test. Chi-squared test and ANOVA test were weighted and accounted for NHANES survey design.

HOMA-IR and HOMA-B varied significantly across cotinine categories with the highest HOMA-IR observed in the 3^rd^ quartile of cotinine but the lowest HOMA-B in the 4^th^ quartile of cotinine.

**Fig 2:**
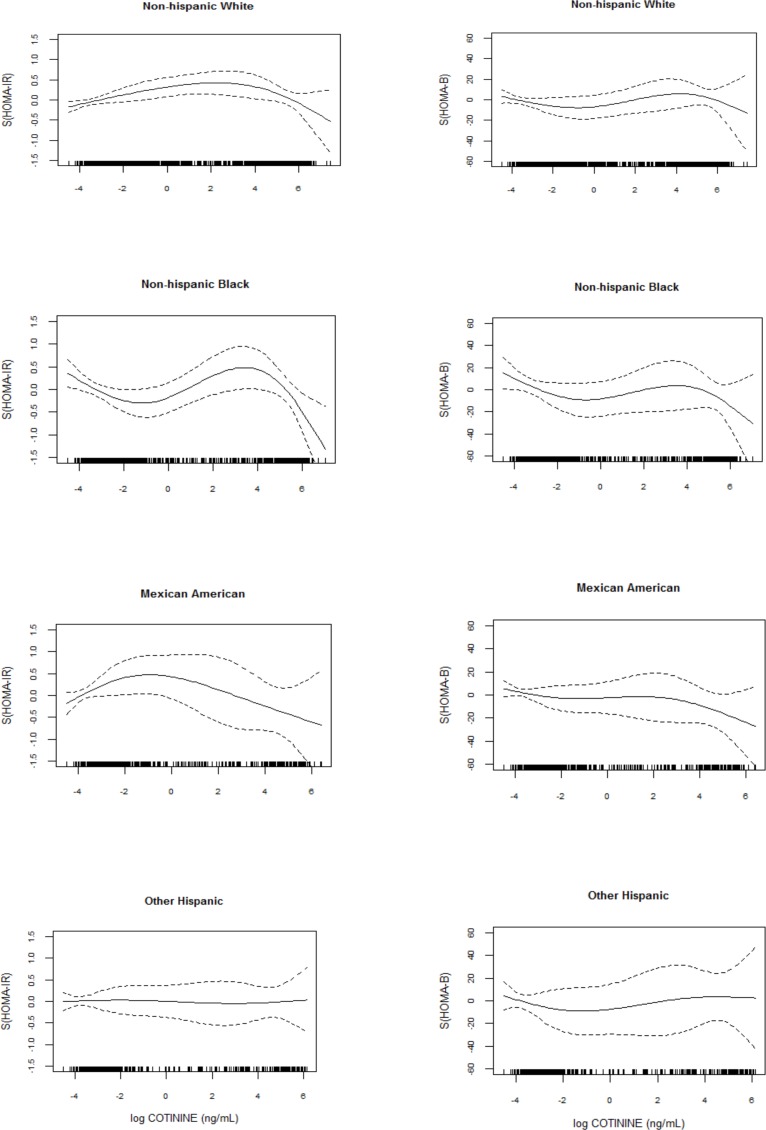
Smoothing plots of HOMA-IR and HOMA-B against cotinine by race/ethnicity.

Spline plots (Fig 2) showed that HOMA-IR and HOMA-B fluctuated with cotinine concentration in a similar shape but varied noticeably by race/ethnicity.

Using logistic regression model, we found no association between cotinine and HOMA-IR among each race/ethnicity participants after fully adjustment (Table 2).

**Table 2:** Associations of quartiles of cotinine with HOMA-IR by race/ethnicity.

	Model 1			Model 2	
Cotinine Quartiles	N of HIR ^a^ (%)	Odds Ratio (95%CI)	P Value	Odds Ratio (95%CI)	P Value
Whole					
1^st^ -2^nd^	696(21.8)	1.00		1.00	
3^rd^	393(26.1)	1.21(1.01–1.45)	0.04	0.96(0.75–1.22)	0.72
4^th^	327(21.6)	0.95(0.77–1.16)	0.60	0.94(0.75–1.18)	0.61
P trend		0.87		0.60	
P^I^		0.53		0.85	
White					
1^st^ -2^nd^	267(20.6)	1.00		1.00	
3^rd^	163(26.6)	1.29(1.00–1.65)	0.04	0.97(0.69–1.36)	0.90
4^th^	184(23.8)	0.94(0.74–1.20)	0.62	0.99(0.74–1.33)	0.82
P trend		0.87		0.34	
Black					
1^st^ -2^nd^	97(26.9)	1.00		1.00	
3^rd^	96(29.6)	1.21(0.81–1.82)	0.38	0.84(0.52–1.35)	0.45
4^th^	73(22.4)	0.84(0.58–1.23)	0.35	0.82(0.54–1.25)	0.23
P trend		0.42		0.92	
Mexican					
1^st^ -2^nd^	97(26.0)	1.00		1.00	
3^rd^	44(29.7)	0.82(0.57–1.19)	0.29	0.90(0.59–1.37)	0.58
4^th^	29(24.2)	0.77(0.43–1.38)	0.38	0.64(0.34–1.21)	0.12
P trend		0.29		0.20	
Hispanic					
1^st^ -2^nd^	182(32.3)	1.00		1.00	
3^rd^	69(30.4)	1.17(0.74–1.84)	0.50	1.07(0.56–2.07)	0.73
4^th^	35(28.5)	1.11(0.65–1.89)	0.70	0.99(0.47–2.07)	0.85
P trend		0.61		0.98	

^a^, high HOMA-IR was defined as HOMA in 4 th quartile.

P^I^, p value for interaction by race/ethnicity.

Model 1, adjusted for age, gender, ethnicity/race, alcohol consumption, education level.

Model 2, Model 1 further adjusted for physical activity and waist circumference.

As shown in Table 3, the risks of low HOMA-B were increased with higher cotinine concentration after adjustment for potential covariates. Yet we observed significant heterogeneity of the association of cotinine with HOMA-B by race/ethnicity, with P values for interactions <0.05 across race/ethnicity groups for each model. Cotinine in 4^th^ quartile was associated with significantly higher risks of low HOMA-B among non-Hispanic White participants (OR, 1.47[95%CI, 1.13–1.91]) and non-Hispanic Black participants (OR, 3.04[95%CI, 1.96–4.72]) compared with the reference group. However, there was no evidence of similar associations among either Mexican American (OR, 1.80[95%CI, 0.92–3.53]) or other Hispanic participants (OR, 1.05[95%CI, 0.95–1.88]). Given the relation of HOMA-IR and HOMA-B, we additionally adjusted for HOMA-IR as well as other covariates included in model 2.The linkage of cotinine with HOMA-B remained significant among non-Hispanic White participants and non-Hispanic Black participants (Table 4).

**Table 3:** Associations of quartiles of cotinine with HOMA-B by race/ethnicity.

		Model 1		Model 2	
Cotinine Quartiles	N of LHB^b^ (%)	Odds Ratio(95%CI)	P Value	Odds Ratio(95%CI)	P Value
Whole					
1^st^ -2^nd^	688(23.9)	1.00		1.00	
3^rd^	309(21.5)	0.89(0.71–1.10)	0.28	1.13(0.89–1.43)	0.35
4^th^	440(30.6)	1.54(1.27–1.88)	<0.0001	1.57(1.28–1.94)	<0.0001
P trend		0.0002		<0.0001	
P^I^		0.003		0.018	
White					
1^st^ -2^nd^	391(30.2)	1.00		1.00	
3^rd^	156(25.5)	0.88(0.65–1.20)	0.43	1.18(0.85–1.66)	0.33
4^th^	248(32.0)	1.48(1.15–1.91)	0.002	1.51(1.16–1.97)	0.002
P trend		0.010		0.004	
Black					
1^st^ -2^nd^	55(15.3)	1.00		1.00	
3^rd^	61(18.8)	1.41(0.87–2.29)	0.17	1.77(1.12–2.80)	0.015
4^th^	100(30.7)	3.01(1.86–4.88)	<0.0001	2.79(1.79–4.69)	<0.0001
P trend		<0.0001		<0.0001	
Mexican					
1^st^ -2^nd^	87(15.5)	1.00		1.00	
3^rd^	41(18.1)	1.09(0.72–1.65)	0.69	1.07(0.64–1.78)	0.80
4^th^	31(25.2)	1.99(1.22–3.24)	0.006	1.79(0.88–3.48)	0.11
P trend		0.016		0.17	
Hispanic					
1^st^ -2^nd^	78(20.9)	1.00		1.00	
3^rd^	23(15.5)	0.81(0.53–1.21)	0.27	0.66(0.41–1.08)	0.10
4^th^	27(22.5)	1.05(0.63–1.78)	0.91	1.06(0.57–1.96)	0.86
P trend		0.99		0.89	

^b^, low HOMA-B was defined as HOMA-B in 4 th quartile.

P^I^, p value for interaction by race/ethnicity.

Model 1, adjusted for age, gender, ethnicity/race, alcohol consumption, education level.

Model 2, Model 1 further adjusted for physical activity and waist circumference.

**Table 4:** Associations of quartiles of cotinine with HOMA-B further adjusted for HOMA-IR by race/ethnicity.

Cotinine Quartiles	N of LHB^b^ (%)	Odds Ratio(95%CI)	P Value
**White**			
**1**^**st**^ **-2**^**nd**^	**391(30.2)**	**1.00**	
**3**^**rd**^	**156(25.5)**	**1.52(1.08–2.14)**	**0.02**
**4**^**th**^	**248(32.0)**	**1.73(1.30–2.32)**	**0.0002**
**P trend**		**0.0001**	
**Black**			
**1**^**st**^ **-2**^**nd**^	**55(15.3)**	**1.00**	
**3**^**rd**^	**61(18.8)**	**1.57(0.80–3.08)**	**0.19**
**4**^**th**^	**100(30.7)**	**2.66(1.54–4.59)**	**0.0004**
**P trend**		**0.0004**	
**Mexican**			
**1**^**st**^ **-2**^**nd**^	**87(15.5)**	**1.00**	
**3**^**rd**^	**41(18.1)**	**1.09(0.72–1.65)**	**0.42**
**4**^**th**^	**31(25.2)**	**2.56(1.03–6.37)**	**0.04**
**P trend**		**0.05**	
**Hispanic**			
**1**^**st**^ **-2**^**nd**^	**78(20.9)**	**1.00**	
**3**^**rd**^	**23(15.5)**	**0.64(0.35–1.16)**	**0.14**
**4**^**th**^	**27(22.5)**	**1.22(0.64–2.31)**	**0.55**
**P trend**		**0.76**	

^b^, low HOMA-B was defined as HOMA-B in 1^st^ quartile.

Adjusted for age, gender, ethnicity/race, alcohol consumption, education level, physical activity, waist circumference and HOMA-IR quartiles.

In sensitive analysis, neither adjustment for antihypertensive use nor exclusion of these participants from the analysis altered our results (data not shown).

## Discussion

### Findings

To our knowledge, no prior study has assessed cotinine-insulin relationships in a large and representative non-diabetic sample. The current study used recent data, representative of the non-diabetic U.S. population, to show that high cotinine concentration is associated with compromised insulin secretion among non-Hispanic White and non-Hispanic Black but not among Mexican American and other Hispanics. There is no association between cotinine concentration and insulin resistance in this population. These findings indicate that compromised insulin secretion but not insulin resistance is operative in the nicotine-diabetes relationship.

### How this study fits into the current literature

Insulin, released by pancreatic β cells, is the most important hormone to modulate glucose metabolism. Defects in insulin secretion and/or insulin resistance are generally considered as the two main pathophysiologic mechanism underlying the development of diabetes[24]. Accumulating lines of epidemic evidence [3, 25] have suggested the association of chronic smoking with diabetes. Yet some clinical studies to explore smoking-insulin relations resulted in ambiguous findings. A case-control study in Sweden found that long-term nicotine-containing gum chewing was associated with insulin resistance[26]. Also, a research of 136 healthy Chinese males[27] observed that smoking was correlated with insulin resistance in term of HOMA-IR. In contrast, Mora-Martinez JM et al [6] failed to detect any influence of transdermal administration on insulin sensitivity in healthy individuals. Although Daniel et al[5] demonstrated the smoking-high β cell function linkage in Canadian, findings from a Sweden study[7] argued that the smoking was associated with lower HOMA-B value. Recently, Clair et al[10] found that cotinine, the biomarker of smoking exposure, was associated with increased chronic glycemia in U.S. population without diabetes. But this study failed to investigate cotinine-insulin relationship underlying the cotininine-glycemia linkage. Our analysis furthered Clair et al’s study to examine cotinine-insulin association by using NHANES data 2007–2012 in non-diabetic American. Not only did we discover that cotinine was associated with impaired insulin secretion in terms of HOMA-B, but also we detected race/ethnicity difference in such associations, which has never been reported in previous studies with small sample size.

The results of the present study are plausible. Quite a few research have discovered neuronal nicotinic acetylcholine receptors (nAChRs) expressed on many different non-neuronal cell types including pancreatic islet cells [28–31]. Basal insulin secretion can be modulated by an endogenous pancreatic ganglionic mechanism. There is evidence that nAChRs are present at the ganglionic level in the pancreas and modulate insulin secretion through a complicated intraganglionic mechanism[32]. Direct evidence of the presence of nicotinic receptors on islet β cells has also been shown, based on mRNA expression [31]. Both long term and acute exposure to nicotine led to a reduction in insulin secretion in response to insulin-secreting agonists, including tolbutamide [31]. Acute exposure to nicotine has been shown to inhibit insulin release at both fasting and high glucose levels [31]. Since functional nicotinic receptors are present in pancreatic islets and β cells and nicotine could partially influence pancreatic β cell function, they may represent a potential switch from which to modulate the physiological function of pancreatic cells in tobacco toxicity, i.e., in smokers/those with high cotinine levels. Other studies [33, 34] have also revealed that nicotine exposure can cause β-cell dysfunction, increased β-cell apoptosis, and loss of β-cell mass, mediated via the mitochondrial and /or death receptor pathway; this suggests other possible mechanisms.

The possible reasons behind the racial heterogeneity include inadequate modeling in one or more racial groups, confounding that differs by race, and biological differences. However, results were robust evaluating race-specific quartiles (S1 Table) and we are unable to identify characteristics that could convincingly lead to differential confounding by race. Therefore, our data indicate that biological differences explain much of the observed heterogeneity. Prior studies[11] have described the racial/ethnic differences in the rate of metabolism of nicotine and cotinine as we observed. At the same daily level of cigarette smoking, higher serum cotinine concentrations are detected in blacks than in whites [35, 36]. And race/ethnicity-specific cut point of cotinine [37] for active smoking also supported race/ethnicity disparity in metabolism of nicotine and cotinine. Such difference may provide clue to the reason for the cotinine-insulin secretion associations only observed in whites and blacks.

### Strengths of our study

First, it is the first time to use cotinine as the surrogate to smoking exposure assessing smoking- insulin linkage. This method avoided potential bias if smokers do not accurately report their smoking status. Second, the large multi-ethnic population sample allowed us to explore the racial/ethnic heterogeneity in the association of cotinine-insulin if exists, which has never been reported in previous studies. Finally, this analysis was conducted in a nationally representative sample; therefore, our results may be generalized to the entire non-diabetic U.S. adult population.

Some limitations must be considered when interpreting our results. Insulin function includes both stable and dynamic stages. In this study we only focused on the stable stage—fasting state, but did not assess the dynamic stages because loaded plasma insulin levels from OGTT were not available in the NHANES data. Moreover, the sample sizes for Mexican and other Hispanics were relatively small and ranges of cotinine concentration were much narrower than other racial/ethnic groups. Further studies on cotinine-insulin with larger sample size are warranted in Mexican and other Hispanic population. Finally, given that this was a cross-sectional analysis, our study cannot make any inferences regarding causality.

In summary, our study indicates that high cotinine concentration/ smoking is associated with compromised stable insulin secretion in a white and black non-diabetic U.S. population. Further longitudinal and experimental research is needed to examine causal relationships from nicotine exposure to β-cell dysfunction and race/ethnicity heterogeneity should be considered when explain the study result.

## Supporting Information

S1 Fig Smoothing plot of cotinine concentration against the average cigarette consumption per day during past month. (DOCX)

S1 Table Adjusted associations of race-specific quartiles of cotinine with HOMA-B by race/ethnicity. (DOC)

S2 Table Least square means of blood pressure and lipid profile stratified by race. (DOCX)

## Abbreviations

HbA1c: Glycated hemoglobin A1c
NHANES: National Health and Nutrition Examination Survey
BMI: Body mass index
WC: Waist circumference
HOMA: Homeostatic model assessment
FPG: Fasting plasma glucose
FPI: Fasting plasma insulin
nAChRs: neuronal nicotinic acetylcholine receptors
