# Supplementary figures and images for: Racial Disparity in the Associations of Cotinine with Insulin Secretion: Data from the National Health and Nutrition Examination Survey, 2007-2012
Source: PLoS One. 2016 Dec 19;11(12):e0167260. doi: 10.1371/journal.pone.0167260 (PMC5167231; doi:10.1371/journal.pone.0167260)

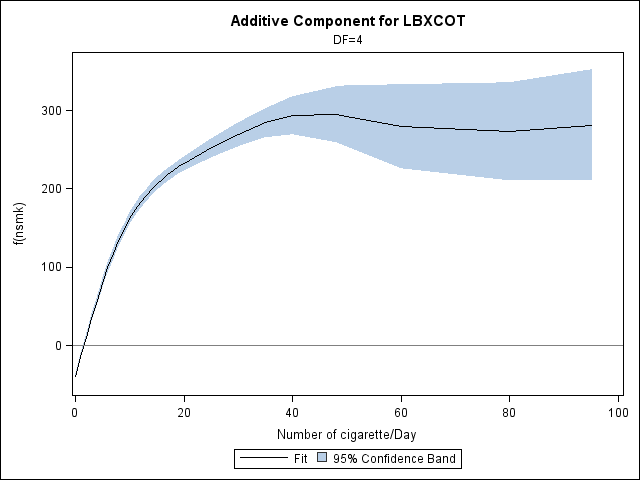

Supplement: S1 Fig — (DOCX) [file pone.0167260.s001.docx]
