# Supplementary material for: Racial Disparity in the Associations of Cotinine with Insulin Secretion: Data from the National Health and Nutrition Examination Survey, 2007-2012
Source: PLoS One. 2016 Dec 19;11(12):e0167260. doi: 10.1371/journal.pone.0167260 (PMC5167231; doi:10.1371/journal.pone.0167260)
Supplement: S1 Table — (DOC) [file pone.0167260.s002.doc]

| **Cotinine** |  | |  | |  | **Model 1** | | **Model 2** | | |  |
| --- | --- | --- | --- | --- | --- | --- | --- | --- | --- | --- | --- |
| **Quartiles** | **Range** | |  | | **N of LHB (%)** | **Odds Ratio**  **(95%CI)** | **P**  **Value** | **Odds Ratio**  **(95%CI)** | **P**  **Value** | | |
| **White** | |  | |  |  |  |  |  | |  |  |
| **1^st^ -2^nd^** | | **0.01-0.05** | |  | **404(30.2)** | **1.00** |  | **1.00** | |  |  |
| **3^rd^** | | **0.05-80.40** | |  | **176(26.1)** | **0.92(0.68-1.26)** | **0.62** | **1.32(0.95-1.84)** | | **0.10** |  |
| **4^th^** | | **81.00-1700.00** | |  | **215(32.0)** | **1.53(1.18-2.00)** | **0.002** | **1.57(1.16-2.11)** | | **0.004** |  |
| **P trend** | |  | |  |  | **0.012** |  | **0.007** | |  |  |
| **Black** | |  | |  |  |  |  |  | |  |  |
| **1^st^-2^nd^** | | **0.01-0.15-** | |  | **81(16.0)** | **1.00** |  | **1.00** | |  |  |
| **3^rd^** | | **0.16-96.30** | |  | **53(21.0)** | **1.73(1.22-1.44)** | **0.001** | **1.87(1.26-2.78)** | | **0.002** |  |
| **4^th^** | | **99.30-1136.00** | |  | **82(32.4)** | **2.55(1.46-4.46)** | **0.002** | **2.35(1.36-4.06)** | | **0.002** |  |
| **P trend** | |  | |  |  | **<0.001** |  | **0.002** | |  |  |
| **Mexican** | |  | |  |  |  |  |  | |  |  |
| **1^st^ -2^nd^** | | **0.01-0.03** | |  | **71(15.5)** | **1.00** |  | **1.00** | |  |  |
| **3^rd^** | | **0.03-0.14** | |  | **40(17.5)** | **1.15(0.76-1.73)** | **0.51** | **1.15(0.73-1.82)** | | **0.52** |  |
| **4^th^** | | **0.14-597.00** | |  | **48(21.1)** | **1.49(0.92-2.24)** | **0.11** | **1.31(0.70-2.46)** | | **0.49** |  |
| **P trend** | |  | |  |  | **0.10** |  | **0.46** | |  |  |
| **Hispanic** | |  | |  |  |  |  |  | |  |  |
| **1^st^ -2^nd^** | | **0.01-0.03** | |  | **72(22.5)** | **1.00** |  | **1.00** | |  |  |
| **3^rd^** | | **0.04-0.26** | |  | **20(12.4)** | **0.41(0.25-0.66)** | **<0.001** | **0.42(0.24-0.74)** | | **0.003** |  |
| **4^th^** | | **0.31-422.00** | |  | **36(22.5)** | **1.05(0.59-1.85)** | **0.87** | **1.12(0.57-2.19)** | | **0.74** |  |
| **P trend** | |  | |  |  | **0.86** |  | **0.96** | |  |  |
| High HOMA-IR was defined as HOMA in 4 th quartile with adjustment for age, gender, ethnicity/race, alcohol consumption, education level , physical activity, and WC. P^I^ , p value for interaction by race/ethnicity. | | | | | | | | | | |  |
