# Supplementary material for: Racial Disparity in the Associations of Cotinine with Insulin Secretion: Data from the National Health and Nutrition Examination Survey, 2007-2012
Source: PLoS One. 2016 Dec 19;11(12):e0167260. doi: 10.1371/journal.pone.0167260 (PMC5167231; doi:10.1371/journal.pone.0167260)
Supplement: S2 Table — (DOCX) [file pone.0167260.s003.docx]

| **Clinic index** |  | **White** | **Black** | **Mexican** | **Hispanic** | **P value*** |
| --- | --- | --- | --- | --- | --- | --- |
| **Systolic pressure (mmHg)** |  | **119.5** | **125.1** | **120.2** | **120.4** | **<0.0001** |
| **Diastolic pressure (mmHg)** |  | **68.9** | **71.4** | **68.6** | **69.5** | **<0.0001** |
| **Total cholesteral (mg/dL)** |  | **195.7** | **193.3** | **199.0** | **197.0** | **0.053** |
| **HDL**  **(mg/dL)** |  | **54.0** | **58.4** | **52.2** | **51.7** | **<0.0001** |
| **LDL**  **(mg/dL)** |  | **116.0** | **116.4** | **119.7** | **119.6** | **0.035** |
| **Tryglyceride**  **(mg/dL)** |  | **131.8** | **93.5** | **139.2** | **129.9** | **<0.0001** |
| *Adjusted for age, waist circumference and gender. | | | | | | |
